# Single-cell transcriptomic characterization reveals the landscape of airway remodeling and inflammation in a cynomolgus monkey model of asthma

**DOI:** 10.3389/fimmu.2022.1040442

**Published:** 2022-11-10

**Authors:** Yingshuo Wang, Xinyan Dong, Caizhe Pan, Cihang Zhu, Hantao Qi, Yifan Wang, Hao Wei, Qiangmin Xie, Lei Wu, Huijuan Shen, Shuxian Li, Yicheng Xie

**Affiliations:** ^1^ Department of Pulmonology, The Children’s Hospital, National Clinical Research Center For Child Health, Zhejiang University School of Medicine, Hangzhou, China; ^2^ Key Laboratory of Respiratory Drugs Research, Zhejiang University School of Medicine, Hangzhou, China; ^3^ The Second Affiliated Hospital, Zhejiang University School of Medicine, Hangzhou, China

**Keywords:** monkey (*Macaca fascicularis*), asthma, scRNA-seq, airway remodeling, inflammation

## Abstract

Monkey disease models, which are comparable to humans in terms of genetic, anatomical, and physiological characteristics, are important for understanding disease mechanisms and evaluating the efficiency of biological treatments. Here, we established an *A.suum*-induced model of asthma in cynomolgus monkeys to profile airway inflammation and remodeling in the lungs by single-cell RNA sequencing (scRNA-seq). The asthma model results in airway hyperresponsiveness and remodeling, demonstrated by pulmonary function test and histological characterization. scRNA-seq reveals that the model elevates the numbers of stromal, epithelial and mesenchymal cells (MCs). Particularly, the model increases the numbers of endothelial cells (ECs), fibroblasts (Fibs) and smooth muscle cells (SMCs) in the lungs, with upregulated gene expression associated with cell functions enriched in cell migration and angiogenesis in ECs and Fibs, and VEGF-driven cell proliferation, apoptotic process and complement activation in SMCs. Interestingly, we discover a novel Fib subtype that mediates type I inflammation in the asthmatic lungs. Moreover, MCs in the asthmatic lungs are found to regulate airway remodeling and immunological responses, with elevated gene expression enriched in cell migration, proliferation, angiogenesis and innate immunological responses. Not only the numbers of epithelial cells in the asthmatic lungs change at the time of lung tissue collection, but also their gene expressions are significantly altered, with an enrichment in the biological processes of IL-17 signaling pathway and apoptosis in the majority of subtypes of epithelial cells. Moreover, the ubiquitin process and DNA repair are more prevalent in ciliated epithelial cells. Last, cell-to-cell interaction analysis reveals a complex network among stromal cells, MCs and macrophages that contribute to the development of asthma and airway remodeling. Our findings provide a critical resource for understanding the principle underlying airway remodeling and inflammation in a monkey model of asthma, as well as valuable hints for the future treatment of asthma, especially the airway remodeling-characterized refractory asthma.

## 1 Introduction

Asthma is a respiratory disease characterized by chronic inflammation and remodeling of the airways, as well as hyperresponsiveness of the airways (AHR) (1). The majority of asthma patients benefit from a combination of inhaled corticosteroids (ICS) and bronchodilators (2). However, approximately 5–10% of patients have refractory asthma and are unresponsive to standard inhaled treatments. One of the explanations for the mechanism of asthma is supposed to be associated with dysregulated innate immunity and Th2-increased inflammation. Immunotherapies, such as anti-IL-5 monoclonal antibodies considered effective treatment strategies (3, 4) with the advent of precision medicine and immune-targeted therapies. For patients lacking type 2 biomarkers, the management of asthma is still an urgent issue (5, 6). Further studies regarding the molecular biological changes and the interaction of different types of cells in the lung tissues in various asthmatic models have great significance for developments in biomarkers and treatments.

Asthma is distinguished by its airway remodeling, which consists of basement membrane thickening, glandular hypertrophy, airway smooth muscle hypertrophy, and new vascular formation. Several studies suggest airway remodeling may be linked to immune and chronic inflammatory responses (7–9). Previous studies indicate that T helper 2 (Th2) cells play a crucial role in airway remodeling in asthma (9). Recent single cell RNA sequencing (scRNA-seq) investigation proved that interleukin-13 (IL-13) is overexpressed in Th2 cells and induces mucin expression and mucus metaplasia in airway epithelial cells (10) and submucosal glands through signal transducer and activator of transcription 6 (STAT-6) activation, leading to airway hyperresponsiveness, mucus overproduction and airway remodeling (8, 11). However, allergic inflammation alone is insufficient to explain the development of asthma. Airway remodeling may exist prior to immune and inflammatory responses (7, 12, 13). Numerous observations have demonstrated that the destruction of airway epithelium in asthma can result in the dysfunction of epithelial cells (7, 14, 15), particularly epithelial-mesenchymal transition (EMT), whose one of the key features is the loss of cell‐cell contact proteins in epithelial cells (2). Airway epithelial injury can result in the release of growth factors (16, 17), including transforming growth factor (TGF), epidermal growth factor (EGF), fibroblast growth factor (FGF), etc. TGF-β induces myoblast differentiation, extracellular matrix (ECM) protein deposition and fibroblast proliferation through intracellular signal transduction mediated by Smad protein (2, 18). Vascular endothelial growth factor (VEGF) promotes angiogenesis and increases the size of airway vessels (16, 19). EGF stimulates mucus secretion by activating the EGFR/Ras/Raf/ERK signaling pathways (20, 21). Currently, the majority of scRNA-seq research is conducted on asthmatic patients and mouse models, which focuses on immune and inflammatory responses (9, 22, 23), but the function and signaling abnormalities of structural cells in airway remodeling need further investigation in various asthmatic models (24).

It is difficult to discern the cellular interactions and molecular mechanisms of airway remodeling in asthma due to the heterogeneity of clinical samples (25) and the homogeneity of mouse models (26). For instance, human ECs can present Ag to resting memory CD4^+^ and CD8^+^T cells, whereas mice ECs can activate CD8^+^T cells but not CD4^+^T (27). It is also difficult to find asthma patients who are not on medication and collect clinical specimens in clinical practice. To better understand the mechanism of asthma development, there is an urgent need for research on highly homogeneous animal models of human asthma (28). Given the difficulties of harvesting untreated clinical asthma samples and other issues, cynomolgus monkeys can be a reliable model for studying asthma and related conditions (29–31). Principally, monkeys share genetic, anatomical, and physiological similarities with humans (32), as well as a susceptibility to asthma. Monkeys and humans have abundant immune and epithelial cells and a similar cell type composition in anatomically identical organs (31). On the other hand, it is possible to avoid the effects of drugs on airway remodeling and to obtain relatively complete samples (33). In addition, there are few studies of scRNA-seq in monkey asthma, but relevant data are urgently required for drug trials and preclinical research.

By establishing an asthma model characterized by inflammation and airway remodeling, we analyzed lung tissues from healthy and asthmatic cynomolgus monkeys at single-cell transcriptomic resolution. Our single-cell analysis identified differences in the proportions and transcriptional phenotype of structural (including stromal cells, epithelial cells and mesenchymal cells) and inflammatory cells in lung parenchyma between normal and asthma groups. The stromal cells expressed genes that represented cell proliferation and migration, such as VEGFA, CTNNB1, and PECAM1 in the asthma group. Besides, we identified a novel subpopulation of stromal cells that differentially expressed POSTN in asthmatic lungs - smooth muscle cell 2 (SMC2), which may contribute to collagen deposition. Further analysis of intercellular communications in healthy and asthmatic airway subtypes revealed a remarkable loss of stromal-mesenchymal cell communication (VEGFA-FLT1) and a concomitant increase between mesenchymal-macrophages cell interactions (TNFRSF1A/1B-GRN), which may relate to increased apoptosis and inflammatory immune responses. Thus, our work generated novel insights into stromal and mesenchymal cell changes and the altered communication patterns between immune and structural cells of the airways that underline asthmatic airway remodeling, which may provide novel approaches for new therapies against asthma.

## 2 Methods and materials

### 2.1 Animals and *Ascaris suum*-induced asthma model

The animal protocol was in accord with the guidelines. A total of fourteen monkeys (crab-eating macaques, 3 - 5 years old) were obtained from the Guangxi Fangchenggang Changchun Biotechnology Development Co., Ltd. Monkeys were raised at a constant temperature of 25°C rooms. Room lighting was automatically controlled on a 12-h light and 12-h dark schedule. Monkeys were fed monkey maintenance feed (purchased from Beijing Keao Xieli Feed Co., Ltd) 100 - 200 g per monkey per day. The managing protocols of the monkeys were carried out in accordance with the standard procedures referring to the Guide for Care and Use of Laboratory Animal (2010) and the principles of Animal Welfare Management (Public Law 99-198). The cynomolgus monkey sample collection and research conducted in this study were approved by the Institutional Animal Care and Use Committee of the Changchun Biotechnology Development Co., Ltd. (Approval Number: 20002).

Monkeys were divided into the control and the asthma model group (four males and three females per group). We performed a skin test on the experimental monkeys before the experiment. All of the experimental monkeys exhibited either no or minimal allergic reaction, showing that these monkeys were not sensitized to *Ascaris suum*. The asthma model group was then sensitized with an injection of 1 mg/mL of *Ascaris suum* (*A.suum*) antigen (34). The process was comprised of 2 separate and consecutive sessions. During session 1, *A.suum*-mixed adjuvant was injected subcutaneously (0.2 mL each site, 1 mL per animal) once every two weeks (weeks 1, 3, and 5) on the back, and inflammation and lung function were measured after antigen challenge. During session 2, asthma models were stimulated with 10 mg/mL of *A.suum* solution intranasally once on weeks 6, weeks 8, weeks 10 and weeks 11 (days 1, 3, and 5 after the last sensitization). The control group was sensitized or stimulated with the same amount of saline. Allergen challenges were performed in 6 and 11 weeks (34). Then, inflammation and lung function parameters were assessed.

### 2.2 Lung function and airway hyperreactivity assessment

Lung functions were assessed before and after the *A.suum*-mixed adjuvant antigen was administered through intermittent positive pressure breathing with a ventilator and in-line nebulizer. Monkeys were anesthetized, and a bundled pressure transducer was connected to a Medlab biosignal acquisition system (U/4C501, MedEase Science and Technology Co., Ltd, USA) to detect changes in the airway flow rates of monkeys after the antigen challenge.

AHR tests were performed 48 h after the last stimulation. Monkeys were administered intravenously by sequential nebulization of 0.15, 0.3, 0.6, 1.2, 2.4, 4.8, 9.6 mg/mL solutions of methacholine (Mch, MKCF3289, Sigma, USA) for 10 times at the each dose. Tidal volume, airway flow rates, and transpulmonary pressure values after nebulizing with Mch were recorded using the Medlab biosignal acquisition system (Medlab-U/4C501, MedEase Science and Technology co.,ltd, USA). The airway resistance (R_aw_) was calculated by transpulmonary pressure/airflow and dynamic pulmonary compliance (C_dyn_) was calculated by tidal volume/transpulmonary pressure.

### 2.3 Tissue dissociation and preparation of single-cell suspensions

Lung tissues were obtained from the control (three males) and asthma groups (three males). After washing in sterile 1× PBS, the tissues were transferred into the culture dish, cut into 0.5 mm^2^ pieces, and washed with 1× PBS to remove blood stains and fatty layers.

The tissues were dissociated into single cells in dissociation solution (0.35% collagenase IV5, 2 mg/mL papain, 120 units/mL DNase I), shaking for 20 min at 100 rpm in a 37 °C water bath. Digestion was terminated with 1× PBS containing 10% fetal bovine serum (FBS), then pipetting 5-10 times with a Pasteur pipette. The resulting cell suspension was filtered by passing through a 70-30 μm stacked cell strainer and centrifuged at 300 g for 5 min at 4°C. The cell pellet was resuspended in 100 μL 1× PBS, added 1 mL 1× red blood cell lysis buffer (130-094-183, MACS, Germany), and incubated at room temperature or on ice for 2-10 min to lyse remaining red blood cells. After incubation, the suspension was centrifuged at 300 g for 5 min at room temperature. The suspension was resuspended in 100 μL Dead Cell Removal MicroBeads (130-090-101, MACS, Germany) to remove dead cells. Then the suspension was resuspended in 1× PBS and centrifuged at 300 g for 3 min at 4°C. The cell pellet was resuspended in 50 μL of 1× PBS. Cell viability was confirmed to be above 85% by trypan blue exclusion using a Countess II Automated Cell Counter (ABI, USA), and the concentration was adjusted to 700-1,200 cells/μL.

### 2.4 Analysis of single-cell data

#### 2.4.1 Single-cell RNA-seq library preparation and sequencing

Single cell suspensions were loaded to 10× Chromium (10× Genomics, USA) to capture more than 3,000 single cells for the Single-cell 3′ Library using Gel Bead Kit V3 (1000075, 10× Genomics, USA) according to the manufacturer’s instructions. The following cDNA amplification and library construction steps were performed according to the manufacturer’s recommendation. Sequencing was performed on the NovaSeq6000 sequencer (Illumina, USA) with a minimum depth of 60,000~90,000 reads per cell and 150 bp (PE150) paired-end reads performed by LC-Bio Technology Co., Ltd (China).

#### 2.4.2 Cell clustering of single-cell RNA-seq data

Sequencing results were demultiplexed and converted to FASTQ format using Illumina bcl2 fastq software v. 2.2.0 (USA). Sample demultiplexing, barcode processing and Single Cell 3’gene counting using the Cell Ranger v. 3.1.0 and scRNA-seq data were aligned to *M.fascicularis* reference genome (macFas6) (31, 32), a total of 35,133 single cells captured from 3 control and 3 asthma monkeys were processed using 10× Genomics Chromium Single Cell 3’Solution. The Cell Ranger output was loaded into Seurat v. 3.1.1 (USA) to be used for Dimensional reduction, clustering, and analysis of scRNA-seq data. Overall, 24,365 cells passed the quality control threshold. To remove the cells with low quality, genes expressed in less than three cell clusters, number of genes expressed per cell > 500 as low and < 5,000 as high, UMI counts less than 500, and the percent of mitochondrial-DNA derived gene-expression > 25% were removed for the following analysis.

To visualize, we further reduced the dimensionality of all 24,365 cells with Uniform Manifold Approximation and Projection (UMAP) for visualization purposes (35). The steps include: Using the LogNormalize method of the “Normalization” function of the Seurat software to calculate the expression value of genes. PCA (Principal component analysis) analysis was performed using the normalized expression value within all the PCs. The top 10 PCs were used to perform clustering and UMAP analysis, then select weighted Shared Nearest Neighbor (SNN) graph-based clustering method to find clusters. Marker genes for each cluster were identified with the Wilcoxon rank-sum test with default parameters *via* the Find All Markers function in Seurat. This selects markers genes expressed in more than 10% of the cells in a cluster and an average log (Fold Change) of greater than 0.25. Violin plots, dot plots and bar plots were drawn by using the ggplot2 package in R. Heat maps were generated by using Heatmapper (36).

#### 2.4.3 Identification of differentially expressed genes (DEGs)

We used the FindMarkers or FindAllMarkers function based on normalized data to identify differentially expressed genes (DEGs). Differential gene analysis was carried out using the “Wilcox” test (37), which returned a p-value using the Bonferroni correction and the log-transformed fold change. Differences with adjusted p-value < 0.05 and log_2_ |fold change| > 0.25 were considered significant.

#### 2.4.4 GO and pathway enrichment analyses

The KEGG pathway enrichment analysis of the differential genes was performed using the ClusterProfiler package. GO enrichment analysis was performed using the OmicStudio tools (https://www.omicstudio.cn/tool/11). Only GO and KEGG terms with a p-value < 0.05 were retained. GO terms associated with near-duplicated terms were removed using a custom script, with the following exclusion criteria, including “POSITIVE”, or “NEGATIVE”.

#### 2.4.5 Construct cell trajectories along the pseudotime

The cell lineage trajectory was inferred using Monocle 2 (version 2.22.0) package according to the tutorial (11). After the cell trajectory was constructed, DDRtree was used to visualize it in two-dimensional space. The UMI matrix was used as input, and variable genes that were detected by Seurat were used for a building trace. For branch site differential genes analysis of MCs, the BEAM function was used to detect genes that contributed most significantly when cells branched.

#### 2.4.6 Cell-to-cell interaction network

To assess the cellular crosstalk between different cell types in lung tissues, we used CellPhoneDB (v2.1.1) with default parameters, a public repository of ligand-receptor interactions (38). The *M.fascicularis* genes were converted to human genes based on homologous gene mapping to run CellPhoneDB analysis. Cell-type‐specific receptor-ligand interactions between cell types were identified based on the specific expression of a receptor by one cell type and a ligand by another. The interaction score refers to the mean total of the average expression values for all individual ligand-receptor partners in the corresponding interacting pairs of cell types, and interactions with a p-value < 0.05 were considered significant.

### 2.5 Quantitative real-time polymerase chain reaction

Total RNA was extracted from lung tissues using TRIzol Reagent (03877, Cwbio, China) according to the manufacturer’s instructions. Total RNA was quantified using a spectrophotometer (Nanodrop 2000, Thermo Fisher, Germany). All the samples presented 260/280 nm ratios between 1.8 and 2.0. Next, cDNA was synthesized using the Prime Script RT Enzyme reagent (AJ10935A, Takara, Japan). Real-time quantitative PCR was performed using the real-time PCR detection system (7500, Applied Biosystems, USA) with TB Green^®^ Premix Ex Taq (AJ61161A, Takara, Japan). The primer sequences of IL-4, IL-5, IL-6, IL-13, CCL17, IFN-γ, TNF-α and β-actin (Sangon Biotech, China) are shown in **Table 1**. The cycling conditions were as follows: 95°C for 10 min, followed by 40 cycles of 95°C for 15 s and 60°C for 60 s. The results were analyzed using the 2^(−ΔΔCt)^ method.

**Table 1:** Primer sequences.

Gene	Sequence #	Primer	Sequence (5’-3’)	
IL-4	XM_015451873.1	F	TCTCACCTCCCAACTGCTTCCC
		R	GTCTTCTGCTCTGTGAGGCTGTTC
IL-5	XM_005557730.2	F	GTGTTGGGCTCCAGTGCTGTG
		R	CTGCGTGGGAATCTGTGTCTGAC
IL-6	NM_001287316.1	F	GGTGTTGCCTGCTGCCTTCC
		R	TGAGATGCCGTCGAGGATGTACC
IL-13	XM_005557731.2	F	GGCAGCATGGTGTGGAGCATC
		R	GGCAGAATCCGTTCAGCATCCTC
IFN-γ	NM_001287657.1	F	CGAATGTCCAACGCAAAGCAGTAC
		R	TGCTCTTCGACCTCGAAACATCTG
CCL17	XM_005592031.2	F	GGACTGTTCCAGGGATGCCATTG
		R	TCTGAGGTGAGGAGGCTTCAAGAC
TNF-α	NM_001285277.1	F	ATGAGCACGGAAAGCATGATCC
		R	CATGGGCTACAGGCTTGTCACT
β-actin	NM_001285025.1	F	CGGGACCTGACTGACTACC
		R	TCCATGCCCAGGAAGGAAG

### 2.6 Analysis of bronchoalveolar lavage fluid samples and serum

Tracheas were inserted with a catheter through an incisal opening sited in the cervical part, and airway lumina were washed. The pooled bronchoalveolar lavage fluid (BALF) was centrifuged and the number of total cells was counted with a hemacytometer. For eosinophils, lymphocytes, and macrophages counting, smears of BALF cells were stained with Wright’s stain and were counted by two independent blinded investigators.

IgE levels were analyzed as 50 μL of serum supernatant combined with 50 μL sample diluent from the ELISA kit according to the manufacturer’s instructions (KA2450, Abnova, China). Following the incubation of allergen-coated wells with a diluted serum sample, the enzyme-labeled reagent was added to each well except for the blank wells. Afterward, the color-developing agent was added to the samples and developed color at RT without light for 10 min. Specific IgE reactivity to the allergens was then estimated by determining the absorbance of each well measured at 405 nm using an automated plate reader. The concentration of IgE was calculated according to the standard curve.

### 2.7 Histology

Lung tissue samples were embedded in paraffin, sliced in 5 μm sections and deparaffinized with xylene following standard procedures. For hematoxylin-eosin (HE) staining, the sections were dried for 3 days at room temperature. Then the tissue sections were placed on glass slides after rehydration and counterstained with hematoxylin before being rinsed with acid water, followed by 10 min wash with tap water. Sections were incubated with Eosin for 5 min, shortly rinsed, dehydrated and mounted with DPX mounting medium.

For Masson’s staining, according to the manufacturer’s instructions (20200528, Solarbio, China), sections were stained with the prepared Harris hematoxylin staining solution for 10 min, incubated in Masson’s blue solution for 5 min and then washed with distilled water. After stained with ponceau magenta solution, they were washed with a weak acid working solution and 1% phosphomolybdic acid solution for 2 min. Then they were washed with prepared weak acid working solution for 1 min and put into aniline blue staining solution for 2 min. Slides were viewed using an Olympus IX71 (Olympus, Japan) microscope, and images were captured using DP controller software.

### 2.8 Immunohistochemical staining

For immunohistochemical (IHC) staining, lung tissues containing large airways were collected from archival formalin-fixed paraffin-embedded blocks. Serial sections (5 μm) were cut using a slide microtome and deparaffinized in xylene. After rehydration, sections were immersed into 10 mM sodium citrate buffer (pH = 6.0). Antigen retrieval was performed by boiling the sections in a pressure cooker at 100°C for 60 min. After washing in PBS, sections were blocked with QuickBlock™ blocking buffer (P0260, Beyotime, China) for immunostaining for 1 h and then were incubated in primary antibody diluted in the blocking solution overnight at 4°C (rabbit anti-α-SMA, 1:100, 55B5-I-AP, Proteintech, USA). After washing, the sections were incubated with corresponding secondary antibodies (Goat anti-Rabbit IgG - 568, 1:1000, A11011, Invitrogen, USA) for 2 h at 37 °C temperature. Then, they were mounted with Dapi containing fluorescence mounting medium solution (C0065, Solarbio, China) after the final wash. The sections were imaged by laser scanning spectral confocal microscopy (TCS SP8, Leica, Germany).

### 2.9 Statistical analysis

For scRNA-seq data, Wilcoxon rank-sum tests were used for statistical significance. P-values were adjusted for the false discovery rate. The genes with adjusted p-value < 0.05 and log_2_ |fold change| > 0.25 were identified as DEGs. For other data, statistical analysis was performed using GraphPad Prism 8.0 (GraphPad Software Inc., USA). Comparisons between the two experimental groups were made using Student’s t-test. p-value < 0.05 and log_2_ |fold change| > 1 were considered to be significant. Data are expressed as the mean ± SE.

## 3 Results

### 3.1 Single-cell RNA-seq analysis of the cell composition of the cynomolgus monkey lung

**Figure 1:**
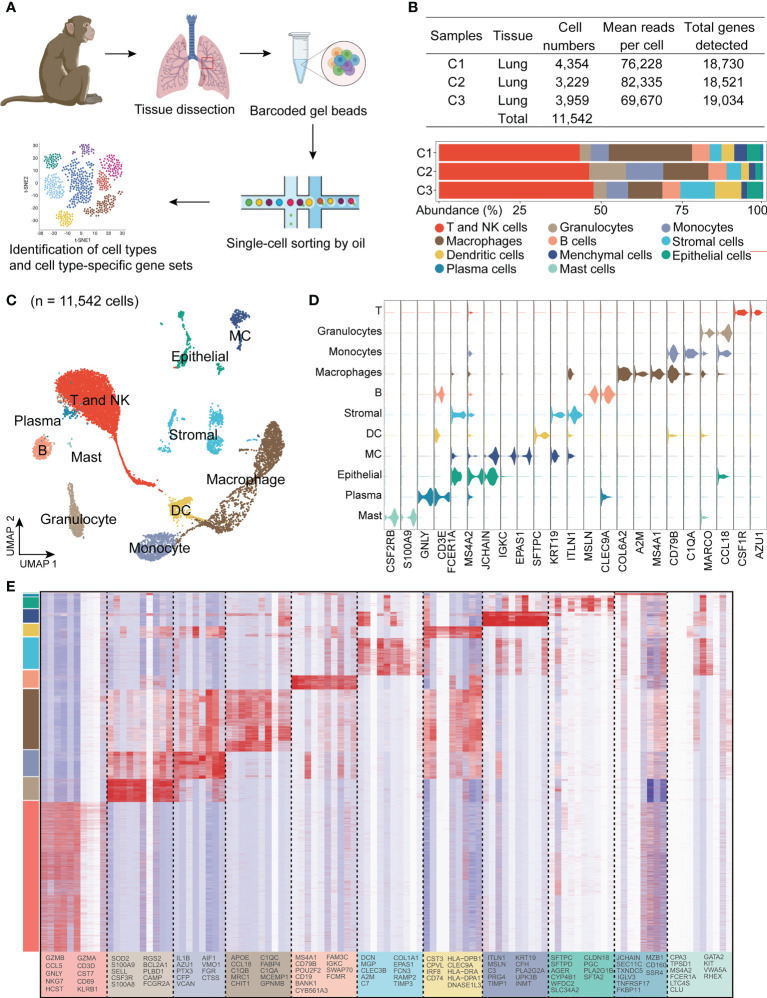
Single-cell RNA-seq identifies eleven major cell clusters in the lungs in healthy cynomolgus monkeys. **(A)**, Experimental design and analysis of single cells from monkey lung samples. The red rectangle indicates an approximate region of sample collection. **(B)**, Table with the details of sample information and representation of major cell subsets as a function of total cells for each sample. **(C)**, Uniform manifold approximation and projection (UMAP) of 11,542 cells from the lungs colored by 11 major cell types based on signature gene expression. **(D)**, Violin plots of unique genes expression specifically distinguishes each cluster (T and NK) (GNLY and CD3E), granulocyte (CSF2RB and S100A9), monocytes (CSF1R, AZU1 and S100A9), macrophage (C1QA, MARCO and Ccl18), B cells (MS4A1 and CD79B), stromal cells (COL6A2 and A2M), dendritic cells (DC) (CLEC9A), mesenchymal cells (MC) (UPK3B, MSLN and ITLN1), epithelial cells (EPAS1, SFTPC and SFTPD), plasma cells (JCHAIN and IGKC) and mast cells (FCER1A and MS4A2), respectively. **(E)**, Heatmap showing row-scaled expression of the ten highest DEGs for all QC-passed cells colored by cell types.

We used 10× Genomics chromium droplet single-cell RNA sequencing (scRNA-seq) to profile 11,542 single cells from large lung airways (**Figure 1A**). We sequenced thousands of cells from each tissue to directly compare cell types without batch correction and did so for three monkeys to address individual differences (**Figure 1B**). High-quality transcriptomes were obtained from approximately 11,542 cells. To gain overall insights into the cellular composition of the normal monkey lung, after normalization of gene expression and PCA, we used shared nearest neighbour unsupervised clustering and UMAP to partition the cells into 11 clusters, including T and NK cells (10,137 cells, marked with GNLY and CD3E), granulocyte (1,563 cells, marked with CSF2RB and S100A9), monocytes (1,838 cells, marked with CSF1R, AZU1 and S100A9), macrophages (4,200 cells, marked with C1QA, MARCO and CCL18), B cells (950 cells, marked with MS4A1 and CD79B), stromal cells (2,522 cells, marked with COL6A2 and A2M), dendritic cells (DC, 804 cells, marked with CLEC9A), mesenchymal cells (MC, 994 cells, marked with UPK3B (39), MLSN (40) and ITLN1), epithelial cells (1,101 cells, marked with EPAS1, SFTPC and SFTPD), plasma cells (154 cells, marked with JCHAIN and IGKC) and mast cells (102 cells, marked with FCER1A and MS4A2) based on their respective molecular features (**Figures 1C–E**).

### 3.2 Inflammatory responses and airway remodeling in the *A.suum-induced* asthma model

**Figure 2:**
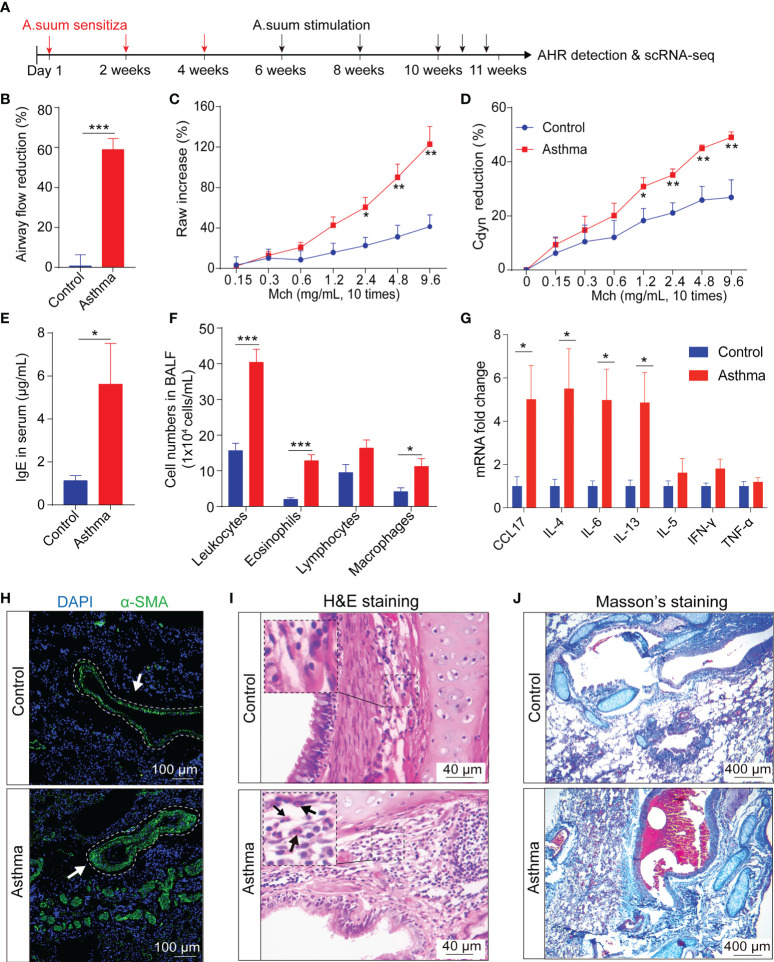
*A.suum* induced asthma results in airway hyperresponsiveness and airway remodeling in cynomolgus monkeys. **(A)**, The experimental design is shown. Lung tissues were collected following AHR assessments. **(B, D),** Airway hyperresponsiveness, including airway flow reduction **(B)**, raw increased **(C)** and cdyn reduction were detected in the asthma group. **(D-F)**, Asthma significantly increases the concentration of IgE **(E)** and the number of leukocytes, lymphocytes, eosinophils and macrophages **(F)** in the BALF. **(G)**, Asthma significantly increases the gene expression related to inflammatory and immunological regulation in the lung compared to the control group. **(H)**, Representative immunofluorescence of interstitial myofibroblast marker α-SMA (green) in airway epithelium, as indicated by white arrows. Scale bar, 100 μm. **(I)**, H&E staining shows infiltration of eosinophils in the airway submucosa. Scale bar, 40 μm. **(J)**, Masson’s staining shows collagen deposition in the lung. Scale bar, 400 μm. ^*^ *p* < 0.05, ^**^ *p* < 0.01, ^***^ *p* < 0.001, Student’s t-test. Data are presented as mean ± SE.

Key features of asthma include chronic inflammation along with airway remodeling. To explore alterations in airway tissue and to investigate the impact on downstream mechanisms of asthma pathogenesis, we used a model of *A.suum*-induced allergic airway inflammation in the monkey. This asthma model accepted *A.suum* stimulation to induce an asthma attack 6 weeks after *A.suum* sensitization, and the airway hyperresponsiveness assay and scRNA-seq were performed in week 11 (**Figure 2A**). These airway function changes include airway flow reduction (*p < 0.001, 58.97 ± 5.55 vs. 0.77 ± 5.61, asthma vs. control group), and abnormal pulmonary airway resistance and dynamic pulmonary compliance. AHR was measured in response to Mch using invasive whole-body plethysmography. When the dose of Mch was 2.4, 4.8 and 9.6 mg/mL, the Raw increase (*p < 0.05, 60.55 ± 9.72 vs. 22.59 ± 8.11; *p < 0.01, 90.16 ± 12.98 vs. 31.32 ± 11.56; *p < 0.01, 122.85 ± 17.63 vs. 41.38 ± 11.65, asthma vs. control group), and Cydn reduction (*p < 0.01, 35.19 ± 2.23 vs. 21.14 ± 3.74; *p < 0.01, 44.96 ± 1.24 vs. 25.87 ± 5.03; *p < 0.01, 49.07 ± 1.99 vs. 26.86 ± 6.49, asthma vs. control group) were significantly higher in the asthma group, which indicates accelerated decline in lung function (**Figures 2B–D**).

IgE contributes to allergic asthma development by modulating the release of mediators from inflammatory cells, thereby initiating the inflammatory cascade. Allergic airway inflammation is mainly accompanied by the release of pro-inflammatory type 2 cytokines. First, we tested the levels of IgE in the serum using an ELISA assay. Increased levels of IgE (*p < 0.05, 4.99 ± 1.69 vs. 1.00 ± 0.21, asthma vs. control group) were observed after administration of *A.suum* (**Figure 2E**). Airway inflammation accompanied by an influx of inflammatory tissue macrophages into the airway spaces. We then analyzed BALF samples using cell counts for inflammatory cells and found elevated leukocytes, eosinophils, and macrophages (leukocytes: *p < 0.001, 40.39 ± 3.67 vs. 15.64 ± 2.07; eosinophils: *p < 0.001, 12.8 ± 1.71 vs. 1.99 ± 0.45; macrophages: *p < 0.05, 11.23 ± 2.17 vs. 4.16 ± 1.07, asthma vs. control group), and a trend toward increased lymphocytes (16.37 ± 2.27 vs. 9.49 ± 2.26, asthma vs. control group), which infiltrated asthmatic airways and collectively promoted exaggerated airway inflammation (**Figure 2F**). Moreover, the levels of the cytokines in the lung tissue after asthma were examined using the qRT-PCR assay. We found that the mRNA expression of CCL17, IL-4, IL-6 and IL-13 was significantly increased in the asthma group (CCL17: *p < 0.05, 5.02 ± 1.55 vs. 1.00 ± 0.44; IL-4: *p < 0.05, 5.51 ± 1.84 vs. 1.00 ± 0.32; IL-6: *p < 0.05, 4.98 ± 1.42 vs. 1.00 ± 0.24; IL-13: *p < 0.05, 4.86 ± 1.39 vs. 1.00 ± 0.28, asthma vs. control group) and a trend toward increased IFN-γ and IL-5 (IFN-γ: 1.81 ± 0.44 vs. 1.00 ± 0.14; IL-5: 1.62 ± 0.65 vs. 1.00 ± 0.24 asthma vs. control group). In contrast, there were no changes of TNF-α levels in asthmatic lung tissues (**Figure 2G**).

Airway remodeling is one of the features of severe asthma. α-SMA is commonly deemed as a marker of released extracellular matrix (ECM) proteins, especially Type I collagen, which is closely related to airway narrowing in asthma (41). Therefore, we further investigated the expression of α-SMA in lung tissues by immunofluorescent staining and found that it significantly increased the expression of α-SMA around the airway smooth muscle compared with the control group (**Figure 2H**). HE staining showed that the *A.suum*-challenged monkey presented abundant infiltrates of peribronchial and perivascular inflammatory cells compared to the control group (**Figure 2I**). Masson’s staining was performed to verify the collagen deposition, which showed more collagen deposition in the airway, perivascular and basement of the asthma group (**Figure 2J**). These data suggest that typical severe airway wall thickening and tissue damage occurred in the asthmatic model.

### 3.3 Cell type-specific transcriptional changes of asthma

**Figure 3:**
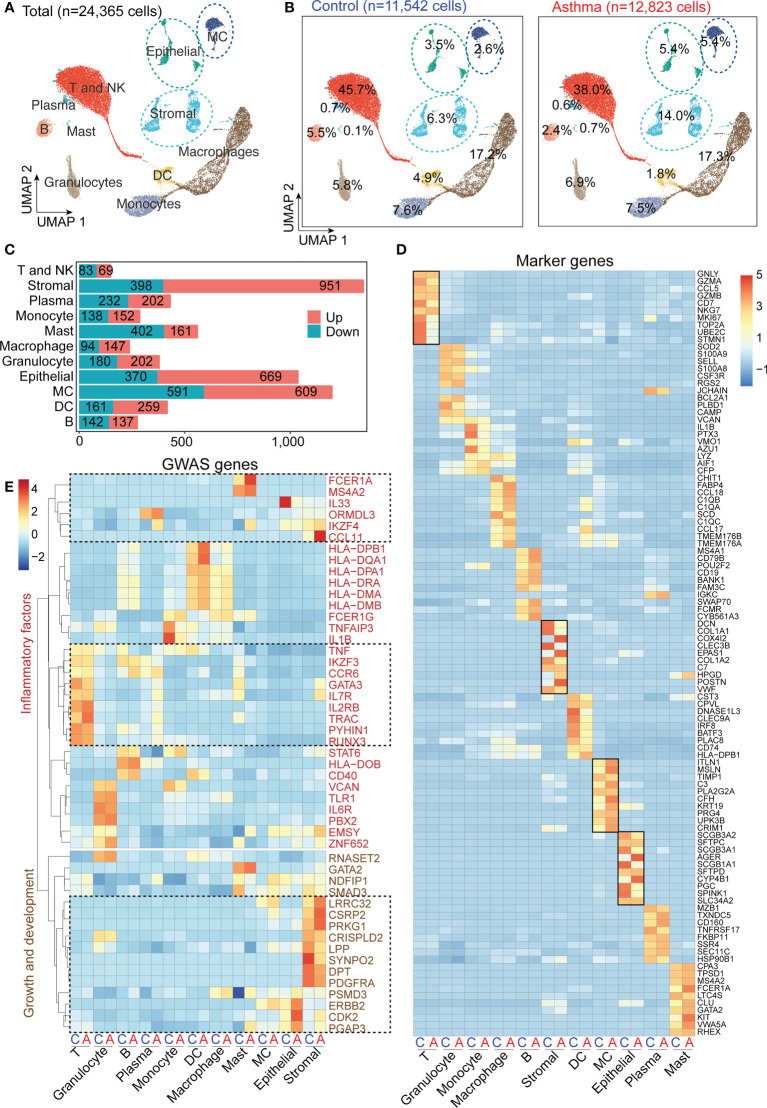
*A.suum*-induced asthma elevates the numbers of stromal and mesenchymal cells and induces various gene expression changes in multiple cell types in cynomolgus monkeys. **(A)**, UMAP of 24,356 cells from asthma and control lungs colored by 11 major cell types based on signature gene expression. **(B)**, UMAP displaying the proportions of cell subsets in the control and asthma group. **(C)**, DEG numbers of control and asthma in 11 major cell types. **(D)**, Heatmap depicting the average expression levels per cluster of the top 10 differentially expressed markers in the control and asthma groups. **(E)**, Heatmap displaying asthma GWAS gene expression per cluster. Only genes enriched in inflammation, growth and development are underlined.

To investigate the cell populations and the associated molecular characteristics in the asthmatic model, three asthma lung samples, and three control lung samples were included in our scRNA-seq analysis. After the removal of low-quality cells, 24,365 cells were retained for biological analysis (**Figure 3A**). We performed statistical analysis on the proportion of 11 cell subsets and the number of differential genes in control and asthma monkeys, respectively (**Figures 3A, B**). Our analysis reveals a significant increase in the proportion of stromal cells (6.3% to 14.0%), MCs (2.6% to 5.4%), and epithelial cells (3.5% to 5.45%) in the asthmatic lungs (**Figure 3B**). Furthermore, we observed a considerable number of DEGs among these three cell subtypes, stromal cells (951 up and 398 down), MCs (690 up and 591 down), and epithelial cells (669 up and 370 down), respectively (**Figure 3C**). Further comparison of the expression of marker genes in all cell subtypes in control and asthma revealed marker genes like DCN, COLOA1, COX4L2, CLEC3B, EPAS1 and POSTN belonging to stromal cells remained the most variable genes (**Figure 3D**). Some DEGs belong to epithelial cells (AGER and CYP4B1), and MCs (ITLN1, MSLN and CFN) were also significantly upregulated. On the contrary, DEGs belong to immunity-related subtypes like T and NK (STMN1, UBE2C and TOP2A), DC (DNASE1L3 and CLEC9A), and macrophages (CHIT1 and SCD) were decreased in the asthmatic model (**Figure 3D**).

After alignment with a genome-wide association study (GWAS) analysis of human asthma genes, which show cell-type restricted expression, we identified multiple factors that potentially affected fibrosis progression (**Figure 3E**) (9). We found stromal cells expressed the highest number of asthmatic GWAS genes, which showed significant differential expression between the control and asthma groups (**Figure 3E**). When characteristics of genes in asthma are divided into two categories: growth and development and inflammatory factors. In our analysis below, we first focused on stromal cells and epithelial cells, and then focused on the MCs and immune compartments.

### 3.4 Specific gene expression signatures of stromal cell subtypes in asthmatic airways

**Figure 4:**
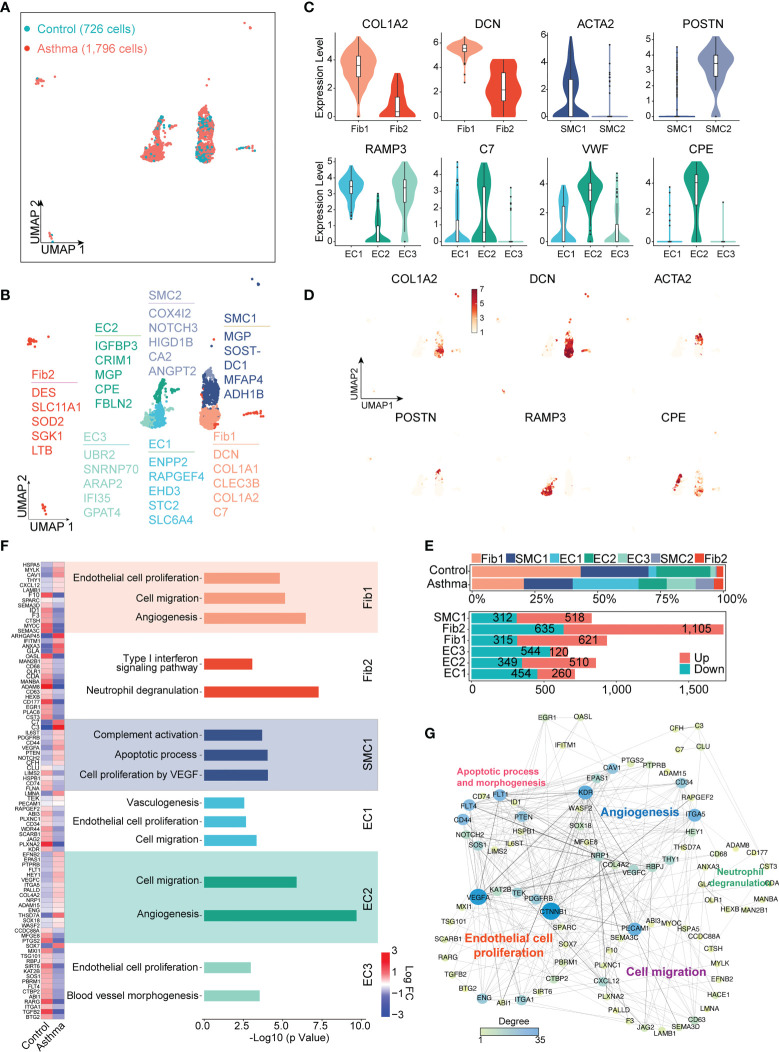
*A.suum*-induced asthma significantly elevates the numbers of ECs, Fibs and SMCs in the lungs of cynomolgus monkeys. **(A)**, UMAP visualization of all scRNA-seq data of stromal cells in the control and asthma groups. Captured cells: control = 726, asthma = 1,796. **(B)**, Seven clusters (subtypes) of stromal cells are colored and labeled according to their inferred cell type identities. **(C)**, Violin plots assessment of signature gene expression in each subtype. **(D)**, UMAP visualization of unique gene expression specifically distinguishes each cluster Fib1 (COL1A2), Fib1 and Fib2 (DCN), SMC1 (ACTA2), SMC2 (POSTIN), EC1 and EC3 (RAMP3) and EC2 (CPE). **(E)**, Representation of the cell numbers proportion and DEG numbers of control and asthma in each subtype. **(F)**, Representation of significant GO and KEGG analysis associated with and heatmap showing the differential genes in biological processes between the control and asthma group. **(G)**, Visualization of gene regulatory network analysis between cell subsets. Interactions between genes are shown as edges. Node sizes and shades of color reflect the degree of centrality and strength of connectivity.

Asthma has the features of chronic inflammation and remodeling of the airway wall. To elucidate the functions of stromal cells in airway fibrosis in asthma, we performed a clustering analysis of the stromal lineage (729 cells in the control group and 1,796 cells in the asthma group) and revealed 7 prominent cell subgroups (ECs 1, 2, and 3, Fibs 1 and 2, as well as SMCs 1 and 2) in asthma versus normal lung parenchyma (**Figure 4A**). EC1 and EC3 were all characterized by high expression of EPAS1, EGFL7, RAMP2, PECAM1 and ESAM, which were involved in blood vessel development and EC proliferation. EC1 and EC3 specifically expressed HPGD, RAMP3, ADGRF5 and ADGRL2. Fib1 expressed higher levels of DCN, COL1A1, and COL1A2 (encodes the collagen fibril forming and assembly), which were also highly expressed in SMC1. Genes involved in tissue development and regeneration and angiogenesis, such as COX4l2, POSTIN, NOTCH3, and HIGD1B were expressed at high levels in SMC2. Fib2 expressed intermediate filament genes, such as DES, and higher levels of immune response (LTB and SLC11A1) and cellular stress response (SGK1 and SOD2) genes compared with the Fib1 (**Figures 4B–D**).

DEGs were also separately identified in subgroups of ECs, SMCs, and Fibs between asthma and control groups (**Figure 4E**). In line with the function of these marker genes, we performed the Gene Ontology (GO) and KEGG analyses for DEGs of subgroups (**Figure 4F**). Statistical analysis of the protein function interaction network suggested that ECs were substantial contributors, particularly to the process of cell migration, proliferation and angiogenesis. KDR ITGA5 and PECAM1 were the central regulatory genes in this protein interaction. Similarly, we also analyzed the putative contributions of SMC and Fib to the progression of asthma. Fib1 also showed some DEGs (e.g., CXCl12 and HSPA5) related to cell migration and proliferation and angiogenesis, similar to ECs. Interestingly, Fib2 exhibited completely different genes and specific pathways than Fib1, including interferon and neutrophil degranulation (e.g., ADAM8, ARHGAP45 and CD177), whose DEGs were also the most pronounced within all subgroups. In contrast, SMCs exhibited a predominance of pathways that regulate complement activation (e.g., C3 and C7) and apoptotic processes. Altogether, these results defined the molecular signatures and functional heterogeneity of various stromal cells, suggesting that the molecular landscape is programmed in the asthmatic model (**Figures 4F, G**).

### 3.5 Typical cell-specific differential gene expression signatures of basal and ciliated subtypes in asthmatic airways

To further understand the subclusters of the critical epithelial cells in asthma, we focused on the epithelial lineage (407 cells in the control group and 694 cells in the asthma group) and their 5 subclusters (AT 1 and 2, basal, club, as well as ciliated cells) in asthma versus normal lung parenchyma (**Supplementary Figure 1A**). We listed the specific markers which were used to define each subcluster (**Supplementary Figure 1B**), and marker genes of each subcluster were mapped on UMAP (**Supplementary Figure 1C**). AT1 specifically expressed CLDN18 and AGER, which were involved in innate immune response and cell adhesion. AT2 was characterized by high expressions of SFTPC, SFTPD, SFTPB (the surfactant secreted by the alveolar cells of the lung), PGC and SLC34A2, which were involved in proteolysis and digestion, and ion transport. Keratin gene families, including KRT5, KRT15 and KRT19, were expressed at high levels in basal. Genes in Club expressed higher levels of cytokine-like protein (SCGB3A2 and SCGB1A1) and immunoglobulin transcytosis (PIGR) genes compared with other subtypes. Ciliated cells expressed cell activation and motility genes, such as DNAH5, TSPAN1 and SPACA9 (**Supplementary Figures 1B, C**). Heatmap displays the normalized expression levels of representative marker genes of each cluster (**Supplementary Figure 1B**).

The proportion of each lung epithelial subtype underwent distinctive changes. The sub-population of basal was dominant in control samples but drastically decreased after asthma (**Supplementary Figure 1D**). To investigate asthma-specific regulation, we performed DEGs analysis for each cell type. Basal and ciliated cells had higher numbers of DEGs (482 up and 313 down, 514 up and 294 down, respectively) compared to other subtypes (**Supplementary Figure 1E**). Next, we performed the GO and KEGG analyses for DEGs of epithelial cells. In the asthma group, basal cells had significant down-regulation of genes (e.g., CXCL10, MMP1 and IL-6) and up-regulation of genes (e.g., MUC5B and IKBKE), which are involved in the IL-17 signaling pathway, accompanied by up-regulation of the apoptosis process (e.g., UBD and AKAP13) and down-regulation of the hippo signaling pathway (e.g., LRG1 and MYC). Increased expression of genes involved in ubiquitin process apoptosis (e.g., DDB2 and INO80) and DNA repair (e.g., USP38 and FBXO22) were observed in ciliated cells (**Supplementary Figure 1F**). Interestingly, we observed that the IL-17 signaling pathway was a common enriched pathway of AT2, club, and basal cells in the asthmatic group (**Supplementary Figure 1F**), and epithelial cell migration, neutrophil degranulation and inflammatory response were enriched in AT1, which suggests that the high relevance of immune response in multiple cell types in the asthmatic model (42). We further constructed a protein function interaction network of specific differential genes, which revealed that the main functions of the core genes in the network include promoting apoptotic response (UBD and FOS), modulating immune response (NF-κb, IL-6, CXCL8 and CSF2), and promoting cell proliferation and migration (CTNNB1 and ACTB, **Supplementary Figure 1G**). Notably, compared to other subtypes, more cell-type-specific genes (UBE2D1 and PSMA2) regulated ubiquitination were enriched in ciliated, highlighting the potential cell-type and function-specific effects (**Supplementary Figure 1G**).

### 3.6 Functional diversity and specific roles of mesenchymal cell subtypes

**Figure 5:**
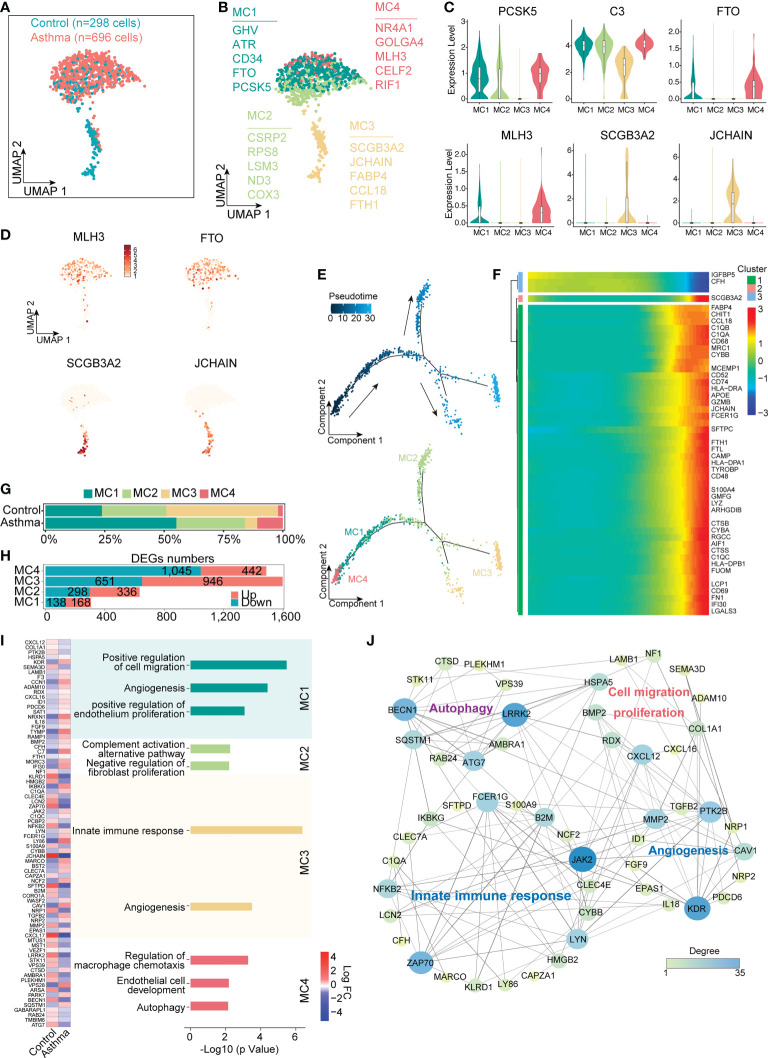
Mesenchymal cells participate in the regulation of airway remodeling and immune response in the lungs of the *A.suum*-induced asthma. **(A)**, UMAP visualization of all scRNA-seq data of mesenchymal cells in control and asthma groups. Captured cells: control = 298, asthma = 696. **(B)**, Four clusters (subtypes) of mesenchymal cells are colored and labeled according to their inferred cell type identities. **(C)**, Violin plots assessment of signature gene expression in each subtype. **(D)**, UMAP visualization of unique gene expression specifically distinguishes each cluster MC1 and MC4 (MLH3 and FTO), MC3 (SCGB3A2 and JCHAIN). **(E)**, Pseudotime developmental trajectory analysis from MC depicting how each of the MC1, MC2, MC3 and MC4 subsets relate to each other. Trajectory directions were determined by biological prior. **(F)**, Heatmap showing scaled expression of dynamic genes along the pseudotime, and these genes were clustered into three groups according to their expression pattern along the pseudotime. **(G, H)**, Representation of the cell numbers proportion and DEG numbers of control and asthma in each subtype. **(I)**, Representation of significant GO and KEGG analysis associated with mesenchymal and heatmap showing the differential genes in biological processes between control and asthma. **(J)**, Visualization of gene regulatory network analysis between cell subsets. Interactions between genes are shown as edges. Node sizes and shades of color reflect the degree of centrality and strength of connectivity.

The number and proportion of interstitial cells also changed significantly in the asthmatic model. Here, we performed sub-clustering of the MC lineage (298 cells in the control group and 696 cells in the asthma group), which revealed 4 distinct groups (**Figure 5A**). Given that transcription factors are crucial in defining cell identity, we next analyzed transcription-factor expression levels in MC1, MC2, MC3 and MC4 (**Figure 5B**). Both MC1, MC2 and MC4 exhibited specific expression of development and cellular differentiation transcription factors (such as PCSK5, GHV and FTO in MC1, PSCK5 and NR4A1, GOLGA4, MLH3 and RIF1 in MC4, **Figure 5B**) but these genes were rarely detected in MC3. MC3 cells exhibited a high expression level of marker genes such as SCGB3A2, JCHAIN, CCL18 and FTH1, which are involved in immune responses (**Figures 5C, D**). We next used the Monocle analysis toolkit to perform cell trajectory analysis to investigate the potential transition between cell types. The pseudotime trajectory axis derived from Monocle indicated that MC4 cells can transdifferentiate into MC1 cells and then into MC3 (**Figure 5E**). Pseudotemporal expression dynamics of specific representative genes also marked the progression of MC4 to MC3 (**Figure 5F**). The results presented here for the first time delineate the potential differentiation paths of MC cells at a single-cell level.

Despite the fact that the difference in the number of cells was not significant, the number of differential genes in each subtype changes significantly in the asthmatic model (**Figures 5G, H**). We next investigated the molecular characteristics change of MC1, MC2, MC3 and MC4. We compared the differential genes of 4 subtypes of cells after the asthma attack and found highly variable genes in MC1 were concentrated in biological processes, including cell migration and proliferation, which were also widely expressed in epithelial cells (e.g., KDR and LAMB1). The typical differential genes concentrated in autophagy-related signaling pathways in MC4 (e.g., BECN1, ATG7 and LRRK2) were found to be downregulated in the asthma group compared with the control group (*p* < 0.05). In contrast, MC3 exhibited a significantly enhanced immune function, which was supported by the upregulation of genes involved in the innate immune response process (e.g., JAK2, C1QA, C1QC and FCER1G, *p* < 0.05, **Figures 5I, J**). These results illustrated that MC4 represented a subpopulation of undifferentiated status, while MC2 and MC3 represented a different subpopulation of differentiated cells with features of mature mesenchymal cells. In addition, MC1 represented an intermediate state. MC1, MC2 and MC4 displayed functional enrichment in biological behaviors related to cell migration, angiogenesis, endothelial cell development and proliferation. By contrast, MC3 exhibited enrichment in signaling pathways such as innate immune responses and angiogenesis (**Figure 5I**). Further analyzing the protein function interaction network of specific differential genes, we found that the main functions of the core genes in the network include promoting fibrosis (LRRK2), inhibiting autophagy regulator gene (BECN1), and promoting cell migration and differentiation (KDR, **Figure 5J**).

### 3.7 Role of immune-related macrophages and T cells in asthma

**Figure 6:**
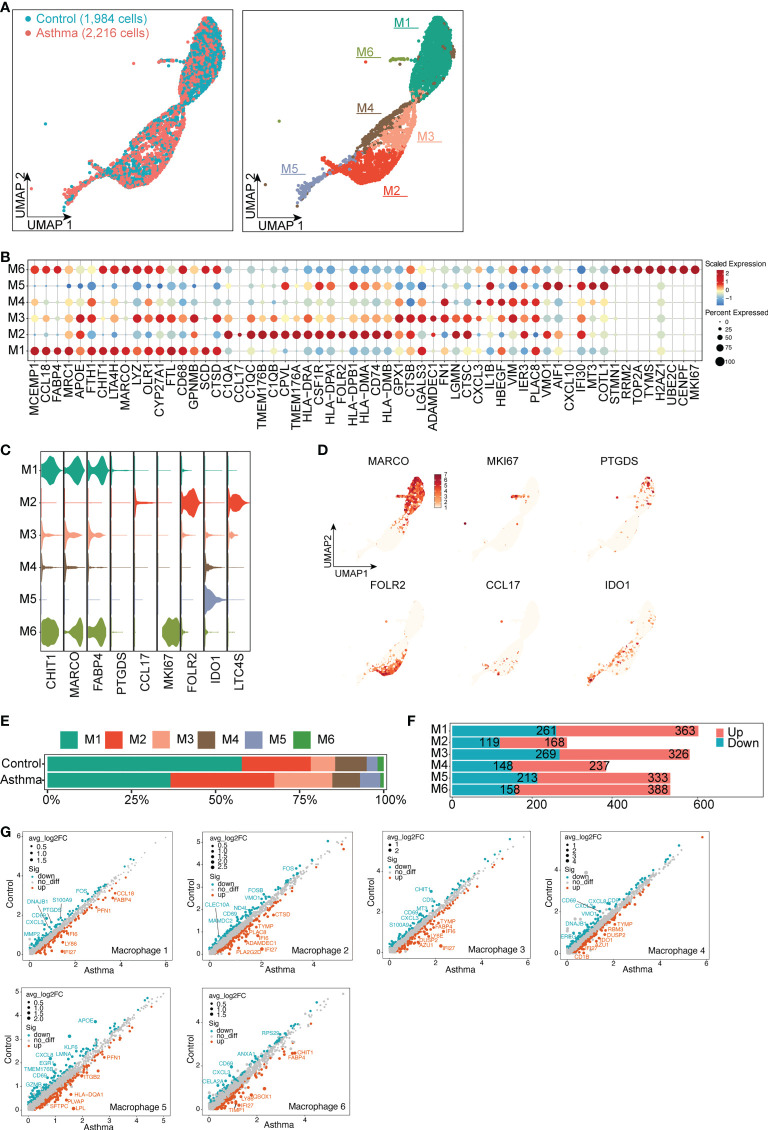
Typical differential genes expression signatures in 6 subtypes of macrophage cells in *A.suum*-induced asthma. **(A)**, UMAP visualization of the macrophages in the control and asthma groups identified 6 subsets. Captured cells: control = 1,984, asthma = 2,216. Each point represents a single cell, colored according to the sub-cluster assigned. **(B)**, Dot plot showing the top ten marker genes in each sub-cluster. Blue and red indicate lower and higher expression, respectively. **(C)**, Violin plots showing the signature gene expression (CHIT1, MARCO, FABP4, PTGDS, CCL17, MKI6, FOLR2, IDO1 and LTC4S) in different macrophage subsets. **(D)**, UMAP visualization of unique gene expression specifically distinguishes each cluster. **(E–F)** Representation of the cell numbers proportion and DEG numbers of the control and asthma in each subtype. **(G)**, Scatter plots showing a pairwise comparison of gene expression between the control and asthma groups. DEGs are highlighted and representative DEGs are labeled. The size of dots is proportional to the fold change of gene expression.

As we aimed to investigate immunological changes in the asthmatic model, we analyzed the single-cell transcriptomes of macrophages and T and NK cells from the large airway of the lungs. We divided macrophages into 6 subsets, M1 specifically expressed MRC1, MARCO and CCL18, which were mainly related to the immunomodulatory effects of macrophages (**Figures 6A–D**). M2 specifically expressed genes related to innate immune response and antigen presentation function (e.g., C1QB, LTC4S, TMEM176B). M3 and M4 specifically expressed FN1 and their genes were mainly involved in the process of fibrosis. M5 specifically expressed COTL1 and AIF1 to promote the production of inflammatory factors and participate in inflammatory responses. M6 specifically expressed genes related to cell proliferation (e.g., MKI67, H2AZ1, STMN1, **Figures 6A–D**). Although there was no significant difference in the number of macrophages, the proportion of each subtype changed in the asthma model compared with the control group (**Figure 6E**). The DEGs in each subtype were compared between the control group and the asthma group by scatter plots. Compared with the other subtypes, the number of up-regulated DEGs was larger in M5 and M6 (333 and 388, respectively, **Figure 6F**). Additionally, the genes involved in proinflammatory function (ITGB2 and FABP4) and apoptosis process (IFI27) were elevated in asthma. By contrast, the immunosuppressive molecule (CD69) was significantly downregulated, which plays key roles in inflammatory conditions of asthma (**Figure 6G**).

Similarly, we grouped T and NK cells and obtained 8 subsets (**Supplementary Figure 2A**). T1 and T5 cell subsets highly expressed genes of cytotoxic molecules (e.g., CD8A, GNLY, GZMA and CCL5), which are mainly involved in CD8^+^ T cell. T1 and T5 also expressed the exhausted CD8^+^ T cell and NK cell marker NKG7, which are supposed to be NKT cells. In contrast, T2 specifically expressed CD4, supposed to be CD4^+^ T cell, which may play an indispensable role in the inflammatory responses in asthma. Among NK cells, 3 subclusters were identified from UMAP. T6, T7 and T8 subtypes specifically overexpressed genes (STMN1, HMGB2, TYMS and MKI67), which are mainly related to cell proliferation (**Supplementary Figures 2B, C**). With regards to the proportions of each T and NK cluster, T2 and T3 subtypes were increased in the asthma model, while the proportion of T6, T7 and T8 subtypes decreased (**Supplementary Figure 2D**). Next, we assessed the contribution of specific T and NK cell subtypes to asthma. Among the DEGs, the number of up-regulated genes in T6 and T8 subtypes were significantly increased, respectively (326 and 348, **Supplementary Figure 2E**). Comparing the 2 groups, GZMB and GZMK were up-regulated in T2 and T3, which are involved in cellular immunity and inflammatory responses. T6, T7 and T8 subtypes upregulated genes (e.g., COTL1, DCN and APOE), which are involved in the inflammatory response (43), fibrosis and lipid metabolism (**Supplementary Figure 2F**). Changes in the number of macrophages, T cells and differential genes suggest that immune cells play an important role in airway remodeling in asthma.

### 3.8 MCs, stromal, and macrophage subsets comprise the core of a predicted cell-to-cell interaction network

**Figure 7:**
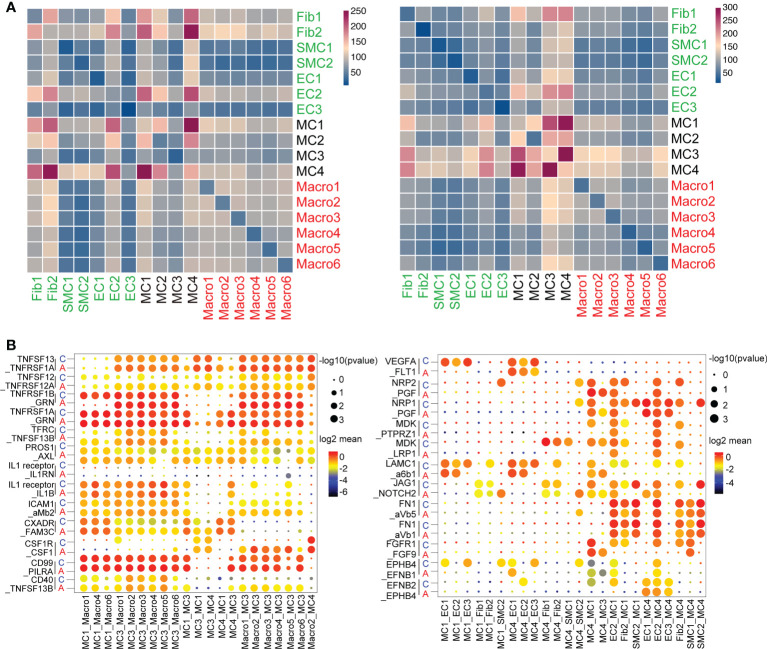
Unique cell-to-cell signaling networks may contribute to the development of asthma in cynomolgus monkeys. **(A)**, Heatmap depicting the number of all possible interactions between all the stromal, mesenchymal and macrophage clusters. Cell types were grouped by broad lineage. **(B)**, Dot plot depicting selected stromal–stromal and stromal–mesenchymal interactions enriched in healthy airways but absent in asthmatic airways (left). Dot plot showing selected mesenchymal–immune interactions highly enriched in the asthmatic airways but absent in the healthy airways (right).

We then constructed a cell-to-cell interaction map by correlating ligands to their corresponding receptors, depicting all altered interactions in asthma compared with the normal group (**Figures 7A, B**). The heatmap represented MCs, including MC1, MC3 and MC4, were more active in communicating with other cell types. (**Figure 7A**). In accordance with previous results, MC1 and MC4 dominated macrophage and structural cell interactions in the control group, whereas MC3 possessed the highest counts of interactions with other cell types, highlighting its central role in cellular crosstalk after asthma (**Figure 7A**).

Despite some minor differences, MCs were still shown to be the major players in cellular crosstalk in asthmatic progression. MC1, MC3 and MC4 displayed altered ligand-receptor interactions with macrophages and stromal cells compared with control, suggesting cell subcluster switching as well as changes in gene expression in the lungs. Analysis of the interaction signaling pathways revealed that MC1 and MC4 interacted with the endothelium through VEGFA, NPR1/2, and MDK-related angiogenesis. The endothelial signaling pathway on MCs was focused on fibronectin1 (FN1) and FGFR1, which were closely related to regulating the fibrosis process. Importantly, top ligands secreted by MC3 were implicated in inflammation (such as TNFSF13 and IL-1 signaling pathway, etc.). The macrophage ligands targeting MC3 were concentrated in innate immune markers, including ICAM and CD40 (**Figure 7B**). Collectively, these observations support that MCs experience substantial changes during the interaction of stromal cells and immune cells that may direct pulmonary fibrosis progression and prognosis in the asthmatic model.

## 4 Discussion

Despite accumulating evidence suggesting that there is cell heterogeneity in the lung, our understanding of structural and functional disorders in the asthmatic model is still hampered by the limited knowledge of cell type complexity, inter-cell type and subtype differences, and cell type- and subtype-specific functions. We described the cellular landscape of cynomolgus monkey lung tissue at the single-cell level and the detailed molecular description of tissue specificity subsets of stomal, epithelial and mesenchymal cells in the airway wall in both normal and *A.suum*-sensitized asthma monkeys. Overall, we provided insights into the cellular composition and interaction networks of the structural cells and immune cells, which further elucidation the roles of different cell subtypes in airway remodeling in asthma.

Here, we established an asthma model using *A.suum*-sensitized cynomolgus monkeys (*Macaca fascicularis*), with asthma symptoms which are consistent with the descriptions in other studies (44), indicating that our model of asthma in monkeys was successfully established. Immunofluorescent analysis revealed subsequent thickening of the airway wall and increased expression of α-SMA (a biomarker of asthma airway remodeling) on the surface of the airway, which suggests subcutaneous fibrosis (45). Moreover, we observed higher concentrations of IgE in the serum of asthmatic monkeys and increased numbers of certain types of immune cells (e.g., lymphocytes, eosinophils, and macrophages) in the BALF, and the elevated mRNA expression of typical immune factor genes (e.g., IL-4, IL-6, IL-13 and CCL17) in lung tissues. Despite the mRNA expression levels of asthma related immune factor genes (INF-γ, TNF-α) showing a rising trend in the asthmatic model (46), suggesting that inflammation plays a significant role in asthma development. Analysis of asthmatic GWAS showed differential inflammatory genes (e.g., HLA-DPB1, HLA-DQA1, FCER1A), which verified the inflammation levels of asthma model. Moreover, genes related to growth and development (e.g., ERBB2, CDK2, PGAP3) were upregulated in asthma, which led to the feature of airway remodeling in the asthmatic monkeys. Overall, these experiments indicate airway remodeling and inflammatory responses as the main features in asthma monkey models.

We applied scRNA-seq analysis of the lungs of cynomolgus monkeys to identify important cell types and functions and to reveal the expression patterns of major genes in different cell types. Airway remodeling in asthma constitutes cellular and extracellular matrix changes, epithelial barrier function, stromal cell proliferation and activation (44). Epithelial cells lose epithelial proteins E-Cadherin in pulmonary inflammatory diseases, which promotes the transformation of epithelial cells into a more mesenchymal phenotype as they acquire mesenchymal markers such as N-Cadherin (47). We observed a significant increase in the proportion of AT1 in asthmatic epithelial cell subsets, and enrichment analysis revealed that DEGs were primarily involved in innate epithelial cell migration, neutrophil degranulation, and inflammatory responses. In the asthma group, the proportion of basal cells decreased significantly, and genes related to mucus secretion (such as MUC5B, et al.) were overexpressed, moreover, which has been proven to promote EMT (48). Besides the classical EMT, we observed an increase in the proportion of Fib and EC in asthmatic lung stromal cells. The overexpression of genes in the type-I-interferon signaling pathway, neutrophil degranulation in Fibs, and cell migration and angiogenesis in ECs may stimulate the production and remodeling of extracellular matrix (49). The highly expressed stromal marker genes like KDR and LAMB1 in mesenchymal cells also indicated the presence of stromal cells in the mesenchyme following an asthma attack. Interestingly, we found a new subtype SMC2 and defined this new cell subpopulation with a relatively increased proportion in the asthmatic model. The special expression genes related to tissue development, regeneration and angiogenesis (POSTN, NOTCH3), suggesting its important role in type-2-mediated eosinophilic airway inflammation and airway remodeling (50). Previous research demonstrated that the alteration of ECs in asthma might result in increased epithelial damage of the airways, thereby promoting airway remodeling (49). We discovered that the expression of some genes was higher in EC1 and EC3 cells than EC2 cells and other subtypes, particularly RAMP3, a type I transmembrane protein, required to transport lung specific protein calcitonin-receptor-like receptor (CRLR) to the plasma membrane (51), which may contribute to the development of asthmatic characteristics. In addition, disruption of the adrenal medulla (ADM - a ligand for RAMP3) is associated with airway smooth muscle hyperplasia and contributes to changes in vascular leakage and epithelial repair in asthma (52). To determine the precise interaction between RAMP3 and ADM that contributes to airway remodeling in asthma, an additional experiment is needed.

Although studies have found a growing appreciation of the importance of epithelial-mesenchymal interactions in airway remodeling (53, 54), the exact role of the mesenchymal compartment in lung homeostasis and disease is still largely unknown. It is essential to precisely determine the taxonomy of lung mesenchymal cells during asthma development by scRNA-seq. Many studies have identified an increased expression of the fibroblast protein (S100A4), matrix metalloproteinases (MMP2 and MMP9) released by myofibroblasts and Fibs can remodel the mesenchymal cells, which play crucial roles in airway fibrosis (55). Our data proved differential genes (MMP2, S100A2) in MC1, MC2 and MC4 exhibited distinct structural cell interactions in the asthmatic model. Moreover, others have demonstrated extensive upregulation of growth factors in epithelial-mesenchymal trophic unit reactivation (17). The physiological effects of VEGFA are mediated by FLT1 and KDR receptors (56, 57). Suppressed VEGF signaling leads to increased levels of SOX9, resulting in mucus metaplasia and excessive secretion (58), which may provide new insights into further understanding of structural cell-related signaling pathways. Our work suggests that MC1 may regulate the normal proliferation of ECs *via* the VGFRA signaling pathway under normal conditions. However, reduced VGFRA-FLT1 signaling may cause destabilized regulation in asthma, resulting in abnormal EC proliferation and endothelial hyperplasia-related gene expression, which increases the susceptibility of the airway to destabilization results in airway remodeling. In addition to their regenerative properties, MCs have strong immunomodulatory effects, including the ability to shift macrophage activation from a pro- to an anti-inflammatory phenotype and inhibit T cell and B cell proliferation (59). We discovered that the TNFRSF1A/1B-GRN signaling between MC3 and macrophages was significantly elevated in asthma, which suggests that MC3 may mediate the coordination of immune responses in the asthmatic model. Relevant studies suggest that macrophages have several contradictory functional roles and are involved in both pro-inflammatory and anti-inflammatory processes. Macrophages can be stimulated and activated into an inflammatory state, which can be roughly divided into two types, M1 (typical activation) or M2 (alternate activation), under appropriate stimulation. The former has been described as proinflammatory effects related to the immune response of bacteria and intracellular pathogens. The latter has anti-inflammatory effects and plays a role in angiogenesis and wound healing (60). In our study, macrophages were divided into six subsets according to different marker genes. Four of these subsets highly expressed proinflammatory genes, such as M2 and M3 (HLA-DR), M4 (IL1B), and M5 (CXCL10 and IL1B), indicating that these subsets promote inflammation. In contrast, M1 and M6 overexpressed an anti-inflammatory marker gene (CCL18). Other studies demonstrated that TNFRSF was mediated by MAPK, NF-κB, and PI3K-Akt signaling pathways, which promoted classically macrophages (pro-inflammatory innate immune functions) to convert to the alternately activated macrophages (functions related to parasite destruction, immune regression, and tissue remodeling) (61). According to our results, the TNFRSF ligand-receptor interaction was significantly elevated between MC3 and macrophages, and differential genes that promote regulation of inflammation (e.g., JAK2, TGFB2 and NFKB2) were also increased in this subtype in the asthmatic model. The precise mechanism may require further investigation. Nevertheless, it can be concluded that the changes in the number and strength of signaling pathways in MC may promote inflammation-related airway remodeling.

Previous research revealed part of the connections between airway remodeling and airway inflammation (7). These changes in airway epithelium in our study are consistent with the study on steroid-resistant asthma mouse models that IL-4, IL-13 and some upstream regulators play an important role in the pathogenesis of asthma (22). Another study of cell census in the human lung suggested changes in the number of different cells and differences in gene expression of cells in the asthmatic airways, identifying Th2 cell-dominated interactions with structural and inflammatory cells, which revealed epithelial-immune and mesenchymal-immune interactions in asthmatic airways (9), being consistent with part of our results. Besides, our studies on structural cells and mesenchymal cells complemented the previous studies and refined the mechanism of asthma. However, there are some distinctions from the previous scRNA-seq results from asthma related studies. First, we used cynomolgus monkeys to establish an asthma model, which is more similar to humans than the mouse model. Compared to the cynomolgus monkey model, the heterogeneity of asthmatic patients was more apparent, and more variables influenced the experimental outcomes. In addition, these studies mostly investigated immune cells *via* cell sorting, whereas research on structural cells was scarce, and the corresponding differential genes and signaling pathways were concentrated among immune cells. Our study employed the whole-cell level, focusing on airway remodeling and intercellular interactions between structural cells, immune cells and other cells, which can reveal the mechanism of asthma in a more comprehensive way. Despite the fact that we discovered relatively significant alterations in the structure of the asthmatic airways, the number and gene expression of mesenchymal cells, and the enhancement of the corresponding signaling pathways, there are still some gaps in the current research. The first limitation is the small sample size: we used a study grouping of 3 monkeys for control and model, respectively. The results revealed substantial individual differences between the monkey asthma models, particularly in terms of total cell numbers. Second, we did not sort the cells prior to single-cell capture, which resulted in a lower total number of detected inflammatory and immune cells, especially eosinophils, which are important in asthmatic airway inflammation. Granulocytes and more detailed subtypes need to be classified and investigated in the following research. Thirdly, the clinical severity of asthma was distinct. The comparison between asthma of varying severity must be further refined in order to reveal the dynamic changes of genes and signaling pathways that occur during the progression of asthma. In addition, further causal functional verification is required for alterations in asthma-related genes and signaling pathways in monkey asthma models.

Through comprehensive analysis of the cell-to-cell interactions, we provide a robust framework for the distinct expression patterns at the single-cell level of interactions between structural cells and other cells in airway remodeling and support the dominance of MC cells interacting with structural and inflammatory cells. The extensive growth factor signaling between stromal cells and mesenchymal cells is largely lost in the asthmatic airway wall, instead of the TNSFR signaling significantly activated between macrophage cells and mesenchymal. In conjunction with the elaboration of the specific mechanisms of immune cells in asthma by the majority of recent studies, our research can effectively complement the structural cell changes for asthma airway remodeling and related intercellular interactions, which are crucial for advancing our understanding of asthma pathogenesis. In addition, airway remodeling is associated with severe asthma (62). We proposed a novel approach based on modifying the genes and intercellular interactions of structural cells involved in asthma airway remodeling in order to identify new anti-asthma drug targets. This may have important implications for the treatment of refractory asthma.

## Data availability statement

The data presented in the study are deposited in the NCBI Gene Expression Omnibus (GEO) repository, accession number GSE213085.

## Ethics statement

The animal study was reviewed and approved by Institutional Animal Care and Use Committee of the Changchun Biotechnology Development Co., Ltd.

## Author contributions

YX, SL, and YSW conceived and designed the study. HW, YFW, CZ, HS, and SL performed the experiments and analyzed the results. XD, CZ, CP, and HQ analyzed scRNA-seq data. YSW, XD, and CP wrote the manuscript. QX and LW participated in the data analysis and interpretation. YX, SL, and HS supervised the project and revised the manuscript. LW, HS, YSW, YX, and SL provided funding support. All authors contributed to the article and approved the submitted version.

## Funding

This work was supported by Grants from the National Natural Science Foundation of China (No. 82173819 to YX, No. 62076218 to YSW, No. 82003760 to HS), the National Key R&D Program of China (No. 2019YFE0126200 to YSW), the Zhejiang Province Public Welfare Technology Application Research Project (No. LGF22H010002 to LW, LQ20H190006 to SL).

## Conflict of interest

The authors declare that the research was conducted in the absence of any commercial or financial relationships that could be construed as a potential conflict of interest.

## Publisher’s note

All claims expressed in this article are solely those of the authors and do not necessarily represent those of their affiliated organizations, or those of the publisher, the editors and the reviewers. Any product that may be evaluated in this article, or claim that may be made by its manufacturer, is not guaranteed or endorsed by the publisher.

## Supplementary material

Supplementary Figure 1 A*.suum*-induced asthma elevates the DEGs of basal cells and ciliated cells in the epithelial cells of the lungs. **(A)**, UMAP visualization of all scRNA-seq data of epithelial cells in the control and asthma groups. Captured cells: control = 407, asthma = 694. Five clusters (subtypes) of epithelial cells are colored and labeled according to their inferred cell type identities. **(B)**, Heatmap showing representative top ten marker gene expression across epithelial subtypes. **(C)**, UMAP visualization of unique gene expression specifically distinguished each cluster AT1 and AT2 (AGER and PGC), Basal (KRT5), Club (SCGB3A2), and Ciliated (DNAH5). **(D–E)**, Representation of the cell numbers proportion and DEG numbers of the control and asthma in each subtype. **(F),** Representation of significant GO and KEGG analysis associated with and heatmap showing the differential genes in biological processes between the control and asthma. **(G)**, Visualization of gene regulatory network analysis between cell subsets. Interactions between genes are shown as edges. Node sizes and shades of color reflect the degree of centrality and strength of connectivity.

Supplementary Figure 2 Typical differential gene expression signatures in 8 subtypes of T and NK cells in the *A.suum*-induced asthma. **(A)**, UMAP visualization of the T and NK cells in the control and asthma groups identified 8 subsets. Captured cells: control = 5,270, asthma = 4,867. Each point represents a single cell, colored according to the sub-cluster assigned. **(B)**, Violin plots showing the signature gene expression (GZMA, OPLAH, THY1, LTB, IL-7R, CD6, SIRPG, STMN1 and HMGB2) in T1-T5 subsets, and (MKI67, TYMS, UBE2C, HELLS and KIF11) in T6, T7 and T8 subsets. **(C)**, Dot plot showing the top 10 marker genes in each subcluster. Blue and red indicate lower and higher expression, respectively. **(D–E)**, Representation of the cell numbers proportion and DEG numbers of the control and asthma in each subtype. **(F)**, Scatter plots showing a pairwise comparison of gene expression between the control and asthma groups. DEGs are highlighted and representative DEGs are labeled. The size of dots is proportional to the fold change of gene expression.
