# Supplementary figures and images for: Single-cell transcriptomic characterization reveals the landscape of airway remodeling and inflammation in a cynomolgus monkey model of asthma
Source: Front Immunol. 2022 Nov 10;13:1040442. doi: 10.3389/fimmu.2022.1040442 (PMC9685410; doi:10.3389/fimmu.2022.1040442)

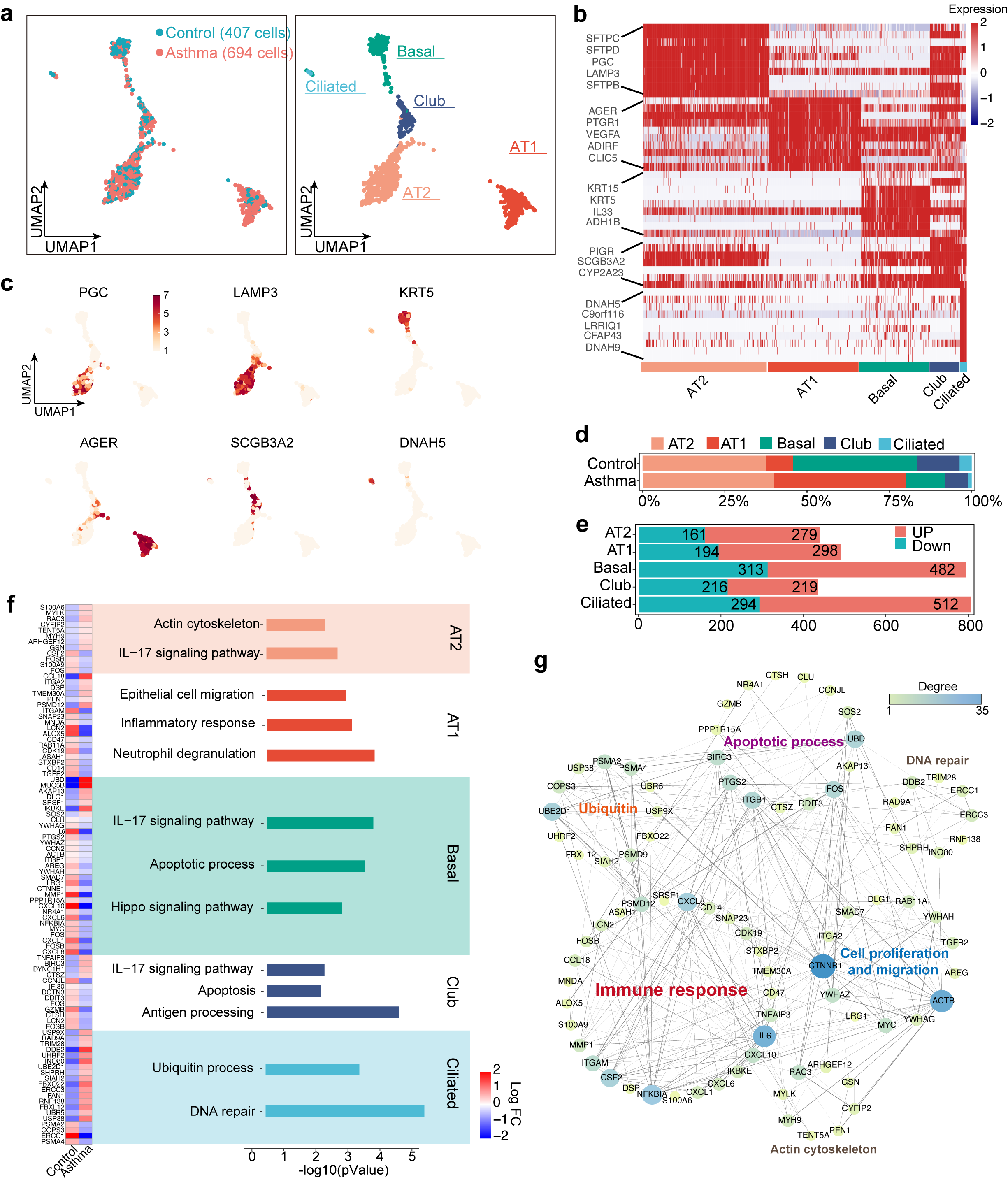

Supplement: Supplementary Figure 1 — A.suum-induced asthma elevates the DEGs of basal cells and ciliated cells in the epithelial cells of the lungs. (A), UMAP visualization of all scRNA-seq data of epithelial cells in the control and asthma groups. Captured cells: control = 407, asthma = 694. Five clusters (subtypes) of epithelial cells are colored and labeled according to their inferred cell type identities. (B), Heatmap showing representative top ten marker gene expression across epithelial subtypes. (C), UMAP visualization of unique gene expression specifically distinguished each cluster AT1 and AT2 (AGER and PGC), Basal (KRT5), Club (SCGB3A2), and Ciliated (DNAH5). (D–E), Representation of the cell numbers proportion and DEG numbers of the control and asthma in each subtype. (F), Representation of significant GO and KEGG analysis associated with and heatmap showing the differential genes in biological processes between the control and asthma. (G), Visualization of gene regulatory network analysis between cell subsets. Interactions between genes are shown as edges. Node sizes and shades of color reflect the degree of centrality and strength of connectivity. [file Image_1.tif]

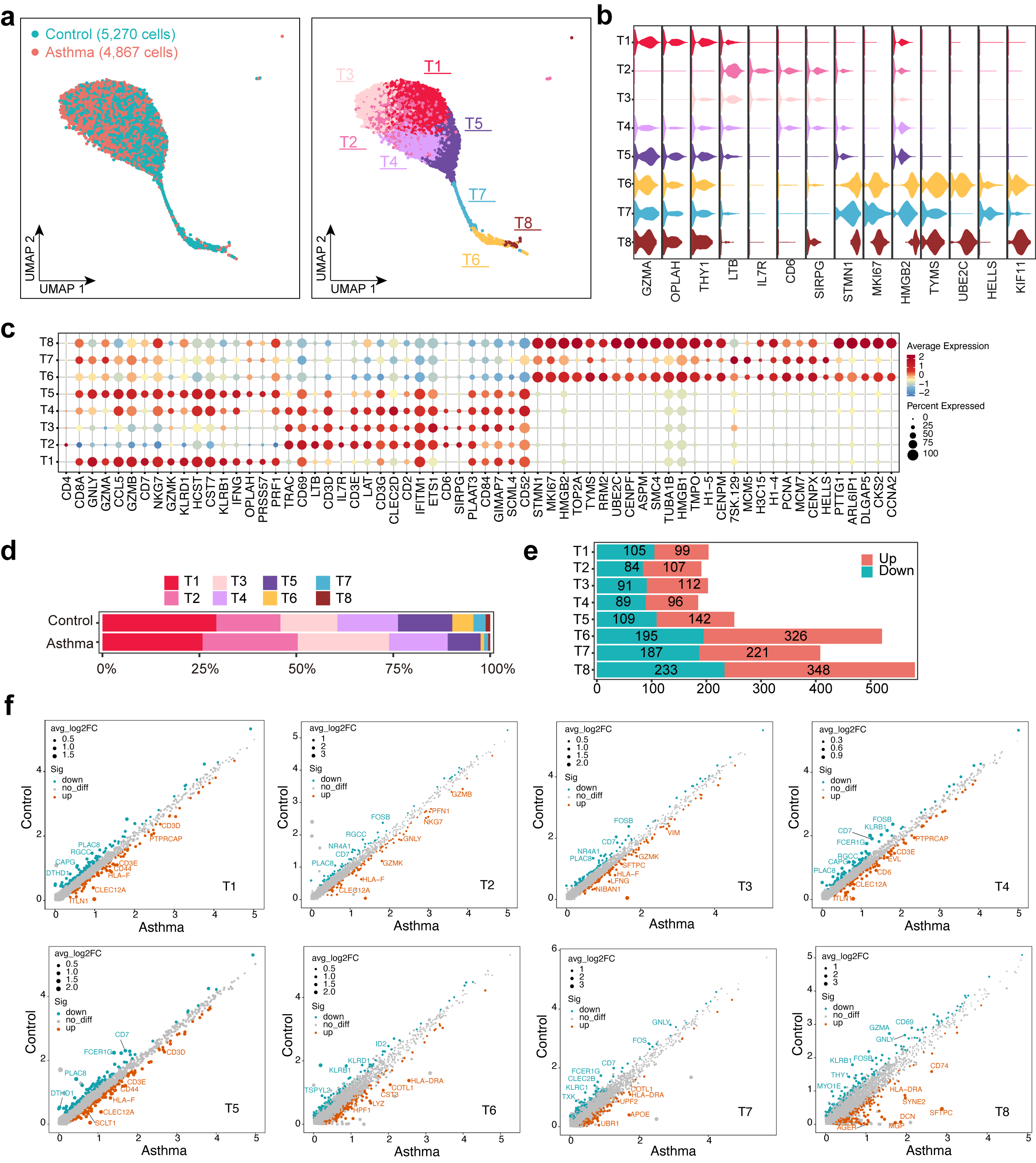

Supplement: Supplementary Figure 2 — Typical differential gene expression signatures in 8 subtypes of T and NK cells in the A.suum-induced asthma. (A), UMAP visualization of the T and NK cells in the control and asthma groups identified 8 subsets. Captured cells: control = 5,270, asthma = 4,867. Each point represents a single cell, colored according to the sub-cluster assigned. (B), Violin plots showing the signature gene expression (GZMA, OPLAH, THY1, LTB, IL-7R, CD6, SIRPG, STMN1 and HMGB2) in T1-T5 subsets, and (MKI67, TYMS, UBE2C, HELLS and KIF11) in T6, T7 and T8 subsets. (C), Dot plot showing the top 10 marker genes in each subcluster. Blue and red indicate lower and higher expression, respectively. (D–E), Representation of the cell numbers proportion and DEG numbers of the control and asthma in each subtype. (F), Scatter plots showing a pairwise comparison of gene expression between the control and asthma groups. DEGs are highlighted and representative DEGs are labeled. The size of dots is proportional to the fold change of gene expression. [file Image_2.tif]
